# Marine-Derived Collagen as Biomaterials for Human Health

**DOI:** 10.3389/fnut.2021.702108

**Published:** 2021-08-24

**Authors:** Ning Xu, Xue-Liang Peng, Hao-Ru Li, Jia-Xuan Liu, Ji-Si-Yu Cheng, Xin-Ya Qi, Shao-Jie Ye, Hai-Lun Gong, Xiao-Hong Zhao, Jiangming Yu, Guohua Xu, Dai-Xu Wei

**Affiliations:** ^1^Department of Orthopedics, Second Affiliated Hospital, Naval Medical University, Shanghai, China; ^2^Key Laboratory of Resource Biology and Biotechnology in Western China, Department of Life Sciences and Medicine, Ministry of Education, School of Medicine, Northwest University, Xi'an, China; ^3^Department of Orthopedics, Tongren Hospital, Shanghai Jiaotong University, Shanghai, China

**Keywords:** marine-derived collagen, tissue engineering, drug delivery system, cosmetics, food, health care product

## Abstract

Collagen is a kind of biocompatible protein material, which is widely used in medical tissue engineering, drug delivery, cosmetics, food and other fields. Because of its wide source, low extraction cost and good physical and chemical properties, it has attracted the attention of many researchers in recent years. However, the application of collagen derived from terrestrial organisms is limited due to the existence of diseases, religious beliefs and other problems. Therefore, exploring a wider range of sources of collagen has become one of the main topics for researchers. Marine-derived collagen (MDC) stands out because it comes from a variety of sources and avoids issues such as religion. On the one hand, this paper summarized the sources, extraction methods and characteristics of MDC, and on the other hand, it summarized the application of MDC in the above fields. And on the basis of the review, we found that MDC can not only be extracted from marine organisms, but also from the wastes of some marine organisms, such as fish scales. This makes further use of seafood resources and increases the application prospect of MDC.

## Introduction

Collagen is a kind of biological macromolecule, which is the richest protein in the human body, accounting for more than 30% of the total body protein (1). It is the main material of extracellular matrix of skin, bone, ligament, cartilage and tendon. More than 85% of human collagen is type I, while other common types of collagens include type II, III, and IV. Collagen is a trimer composed of three polypeptide α chains (2). And it has a typical triple helix structure and glycine, proline and hydroxyproline residues is rich.

Collagen as a biomaterial is widely used in various fields due to its biocompatibility, biodegradability, accessibility and high throughput (3, 4). However, the health of collagen extracted from cattle and pigs is very worrying due to diseases (5). For example, outbreaks of bovine spongiform encephalopathy (BSE), infectious spongiform encephalopathy (TSE) and foot-and-mouth disease (FMD) have aroused wide health concerns about the use of collagen and collagen derived products in terrestrial animals (6). In addition, religious disputes are inevitable (7). At present, collagen has been extracted from many marine products. Marine-derived collagen (MDC) solves the problems of other animal diseases and pathogens. And, MDC has better chemical and physical durability and is abundant in quantity (8, 9).

In recent years, MDC in various fields has been widely used due to its extensive sources, simple extraction methods, good biocompatibility, edibility and so on. This paper summarizes the sources, extraction methods and characteristics of MDC. In addition, the application of MDC in medical tissue engineering, drug delivery, cosmetics, food and other fields was reviewed. On this basis, we preliminarily explored the biocompatibility of gill dolphin collagen and tilapia collagen as well as the application in skin tissue engineering.

Many researchers have been looking for alternative sources of collagen in aquatic animals (10, 11). With the extraction of MDC, fish skin, fish scale and other fishery wastes have been better utilized. Transforming waste into collagen solves the environmental problems related to fish (12, 13). The use of collagen derived from terrestrial animals is controversial due to the problems related to disease, religion and so on. However, as a biomaterial with a wide range of sources, MDC has attracted more and more researchers because of its good biocompatibility and degradation properties.

## Source, Extraction, and Characterization

With the increasing demand for collagen, new materials are needed as the source of collagen (Tables 1, 2). Extracting collagen from marine organisms can not only avoid the problem of religious belief, but also has its unique properties. The efficiency and effectiveness of collagen extraction process has always been considered in the process of collagen extraction. Compared with the conventional acid assisted and pepsin assisted extraction of collagen, the collagen extracted by the improved physical assisted process retains a higher molecular weight, and the peptide spectrum is similar to that extracted only with acid (88). In addition, collagen extracted from dried jellyfish and squid has potential applications in biomedicine, medicine and health care products (89). As shown in Table 3, methods of extracting MDC are reported.

**Table 1:** Characterization and amino acid characteristics of MDC.

**Sources**	**Type**	**Characterization methods**	**Amino acids (composition, content, and characteristics)**	**References**
The skin of Nile tilapia (*O. niloticus*)	Marine collagen peptides	Amino acid analysis	Seven essential amino acids (16.18%) and 10 non-essential amino acids (79.56%); Accounting for over 58% of the total residues in MCPs, were hydrophilic.	(14)
Jellyfish *Rhizostoma pulmo (jCOL)*	Collagen	Biochrome		(15)
Axinella cannabina; Suberites carnosus	Intercellular collagen (ICC)	UV or fluorometry		(16)
Mussel byssus	Collagen	High performance liquid chromatography (HPLC)	Amino acid composition of PSC obtained was similar regardless hydrolysis conditions	(17)
Tra catfish (*Pangasianodon hypophthalmus*), clown knifefish (*Chitala ornata*), and tilapia (*Oreochromis niloticus*)	Acid-soluble collagen (ASC)	Amino acid analysis	glycine 33.2–33.7%; The content of proline and hydroxyproline (imino acid) of collagen from three fish skins is 19.2–20%.	(18)
*Takifugu flavidus*	Collagen	Amino acid analysis	Gly was the most abundant residue; accounting for a quarter of the total amino acid components.	(6)
Eleven fish species inhabiting wide spectrum of temperatures	Acid Soluble Collagends (ASCs)	Circular Dichroism (CD)	Substitution from Hyp to Ser allows greater flexibility in the collagen triple helix; maintaining stability with seryl hydroxyl group driven hydrogen bonds.	(19)
Codfish skin	Collagen	Biochrom	Collagen type I consists of 20 different amino acids organized; three α-chains which wrap around each other; characteristic triple-helix conformation.	(20)
Sturgeon (*Acipenser schrencki × Huso dauricus*)	Type II collagens	Automated amino acid analyzer	The glycine abundant	(21)
Skipjack Tuna (*Katsuwonus pelamis*)	Scale gelatin (TG) and antioxidant peptides (APs)	SDS-PAGE; Fourier transform infrared spectroscopy (FTIR); electrospray ionization mass spectrometers (ESI-MS); radical scavenging assays	TG with a yield of 3.46 ± 0.27% contained Gly (327.9 ± 5.2 residues/1000 residues); content was 196.1 residues/1000 residues; TG was more unstable than that of type I.	(22)
Surf clam shell (*Coelomactra antiquata*)	Collagen	Hitachi L-8800 auto amino acid analyzer (Hitachi, Tokyo, Japan)	Guanidine hydrochloride soluble collagen (GSC) and pepsin soluble collagen (PSC) contained glycine as the major amino acid.	(23)

**Table 2:** Sources of various marine-derived collagen (MDC).

**Species**	**Tissue or organs**	**References**
Tilapia	Skin	(14, 24–27)
	Scale	(28–35)
	Unknown	(36–39)
Jellyfish	Unknown	(40–47)
Shark	Skin	(48–51)
	Cartilage	(52)
Salmon	Skin	(53–59)
	Bone	(60)
	Scale	(60)
Sponge	Unknown	(27, 61–66)
Snakehead fish	Scale	(67)
	Unknown	(68)
Tuna	Skin	(69)
	Unknown	(70)
Others: Prionace glauca		
	Skin	(71)
Giant croaker (Nibea japonica)	Swim bladder	(72)
Sole fish	Skin	(73)
Codfish	Skin & bone	(74)
Sparidae	/	(75)
Sturgeon fish	/	(76)
Gadiformes	Skin	(77)
Mrigal fish	Scale	(78)
Flatfish	Skin	(79)
Weever	Skin	(80)
Seabass	Scale	(81)
Silver carp	Skin	(82)
Synodontidae fish	Scale	(83)
Eel	Skin	(84)
Codfish	/	(20)
Gadus morhua	/	(85)
Cyprinus Carpio	/	(86)
Grouper	Swim bladder	(87)

**Table 3:** Extraction methods of MDC and their advantages and disadvantages.

**Sources**	**Extraction method**	**Principle**	**Advantages**	**Disadvantages**	**References**
Mussel byssus	Pepsin solutions	Pepsin is typically indiscriminate in its digestion of proteins, with the notable exception of the triple helical domain of native collagen with further limited pepsin digestion, the cross-linked molecules at the telopeptide region are cleaved without damaging the integrity of the triple helix.	•Ensure the integrity of the collagen molecule		(17)
Axinella cannabina; Suberites carnosus	Alkaline solubilization, trypsin solubilization	The first method was initially introduced for the isolation of insoluble collagen (InSC) from *G. cydonium* and *C.reniformis* by employing an alkaline, both denaturing and reducing, homogenization buffer affording collagen in high yield;The second one utilizes a trypsin-containing extraction buffer, known to destroy the interfibrillar matrix and, therefore, releasing the collagen fibrils (ICC). After exhaustive water extraction, the remaining debris generally comprises the spongin/spongin-like collagen.		•Reagent residues in collagen; •Generate abundant waste liquid; •Resulting in environmental pollution	(16)
Surf clam shell (*Coelomactra antiquata*)	Guanidine hydrochloride and pepsin		•Safer; •Cheape; •More moderate; •Less destructive than acid hydrolysis		(23)
Indian major carp rohu (Labeo rohita)	Enzymatic method				(90)
Bigeye tuna	Acetic acid and pepsin				(70)
Shark (Prionace glauca) and ray (Zeachara chilensis and Bathyraja brachyurops)	Acidic and enzymatic extractions				(91)
Codfish skin	An acid-base procedure			•Ineffective with byssal threads	(20)
Salmon Byproducts	Bacterial extracellular proteases fermentation	The proteases secreted by marine bacteria play an important role in the decomposition of organic nitrogen in oceans.	•Potential bioactive peptides would be released; •The reaction time is shortened.		(92)
Nile tilapia (*Oreochromis niloticus*) skin	Collagen extraction after fermentation pretreatment		•Type I collagen with high purity; •Retained the integrity of their triple helical structure.		(93)
Jellyfish (Acromitus hardenbergi)	Physical-aided acid-assisted extraction method	Increase physical intervention.	•Similar amino acids composition; •Retained high molecular weight distributions;		(88)
*Takifugu flavidus*	Electrodialysis extraction	This method can purify charged proteins/peptides by ion-exchange membranes through a stimulated diffusion process under the influence of electric potential difference.	•High efficiency; •Large capacity; •High extraction yield; •Better environmental sustainability		(94)
	Freeze drying and electrospinning processes				(40)
Tilapia	Electrospinning		•Simple operation		(36)

Electrodialysis is also a promising technology, but it has not been applied to the extraction of fish collagen. At present, the physical and chemical properties of flavonoid collagen are retained by electrodialysis, which fully shows its advantages in the experiment. Therefore, we can assume that electrodialysis can also improve the production environment of fish collagen (95). The extraction of collagen from fish skin improves the value of marine by-products and avoids the pollution caused by large amounts of waste. Taking Atlantic cod as an example, the extraction rates of collagen by acetic acid and pepsin were 5.72 and 11.14%, respectively (96). Compared with the traditional organic acid solution extraction, the extraction rate of collagen and the properties of products are improved by CO_2_ acidification water, which has potential value in the field of biomedicine and cosmetics (97).

**Figure 1:**
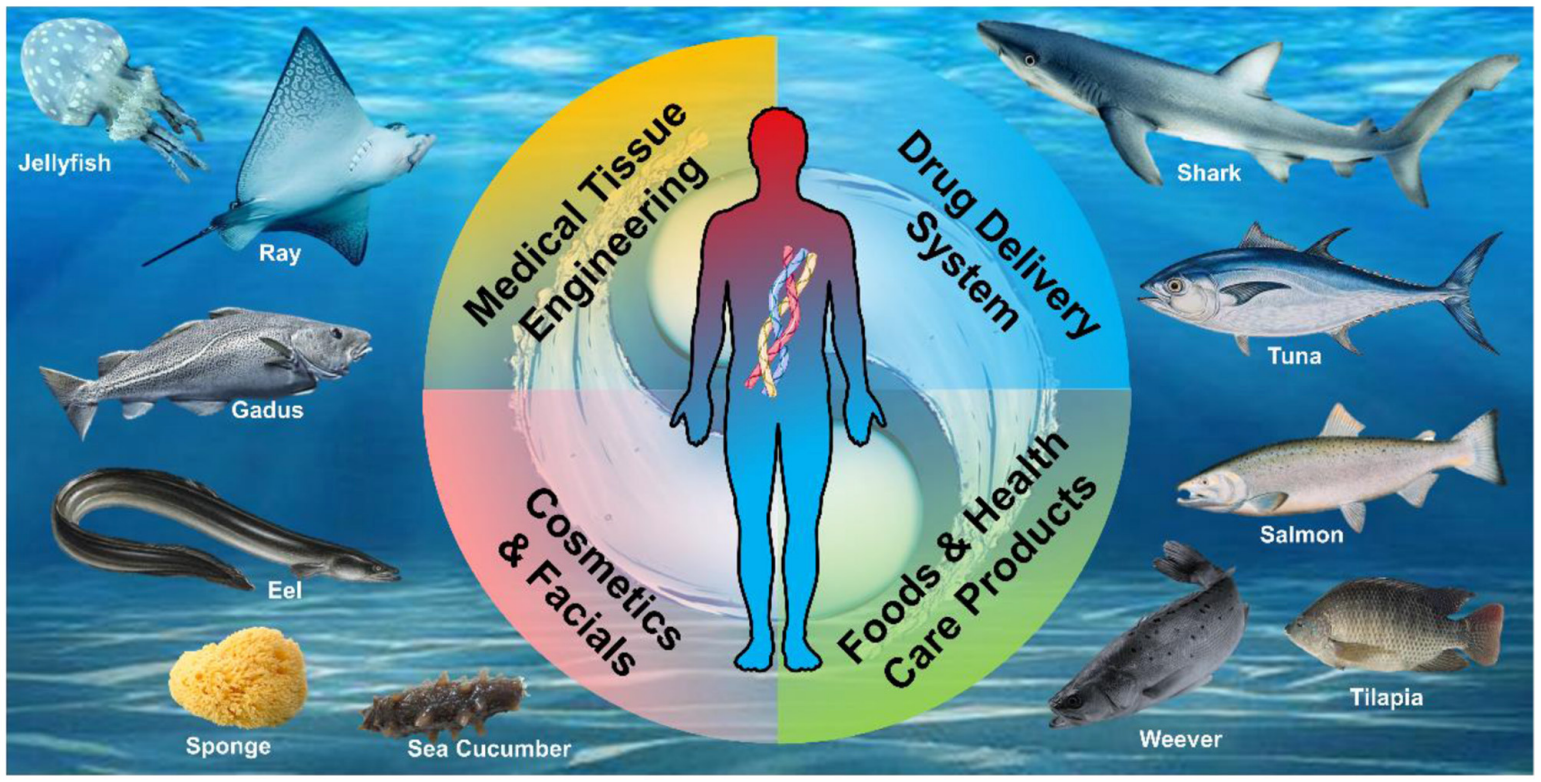
Abundant marine-derived collagen (MDC) as biomaterials are extracted from various marine organisms for human health.

Marine resources have great potential (Figure 1). When looking for natural moisturizing cosmetics, sea cucumber is finally selected. Pepsin soluble collagen was extracted from sea cucumber wall. Its moisture retention and moisture absorption with tilapia collagen are better than those of glycerol, which shows the potential application of MDC in cosmetics (98) (Table 4).

**Table 4:** Applications of MDC in medical tissue engineering.

**Applications**	**Manufacture technique**	**Forms**	**Additive materials**	**Biological evaluation**	**References**
BoneTissueEngineering	/	Scaffolds	Hydroxylapatite	/	(99)
	/	Scaffolds	Hydroxylapatite	/	(100)
	Freeze-drying and EDC cross-linked	Scaffolds	Alginate	hMSCs	(41)
	/	Solution	Moringa oleifera	Albino rats	(101)
	Freeze-drying/dehydrothermal treatment	Scaffolds	Glycosaminoglycan	MC3T3-E1	(48)
	/	Solution	/	Mouse	(49)
	Hydrolysis	Solution	/	BMSCs	(102)
	/	solution	/	Human osteoblasts	(74)
	/	Scaffolds	/	Wistar rats	(66)
	/	Peptide solution	Calcium aspartate	Rats	(103)
	Freeze-drying	Native collagen	/	Primary hMSCs	(28)
	Enzymatical hydrolysis	Peptide	/	Rats	(51)
	Enzymatical hydrolysis	Peptide	/	MG-63 cells	(75)
	Freeze-drying/EDC cross-linked	Scaffolds	/	NIH3T3, MG-63 cells and Mouse	(104)
	Freeze-drying/EDC/NHS or HMDI cross-linked	Scaffolds	/	Saos-2 cells	(50)
	Freeze-drying	Scaffolds	Chitosan/Hydroxyapatite	MG-63 cells	(61)
	Freeze-drying/Glutaraldehyde cross-linked	Scaffolds	Chitosan/Hydroxyappatite	6T-CEM cells	(52)
	Vacuum drying	Scaffolds	Hydroxyapatite/PMMA	MC3T3-E1 cells and L929 cells	(62)
	Glutaraldehyde cross-linked	Scaffolds	Poly (N, N'-dimethylacrylamide)	Rabbit bone defect model	(76)
	Electrospinning	Scaffolds	PLGA/Hydroxyapatite	Primary BMSC and Human gingiva fibroblasts	(6)
Cartilage tissue engineering	Freeze-drying/Chemical cross-linking.	Scaffolds	/	hMSCs	(105)
	Freeze-drying	Scaffolds	/	Rabbit	(106)
	Freeze-drying	Collagen solution	/	hMSCs	(42)
	Freeze-drying	Scaffolds	/	Rabbit chondrocytes and Rude mice	(107)
	Cryogelation	Scaffolds	/		(71)
	Freeze-drying	Peptide solution	/		(91)
	Enzymatical hydrolysis	Peptide	/	Primary horse adipose-derived stromal cells	(108)
	Enzymatical hydrolysis	Peptide	/	Rabbit osteoarthritis model	(77)
	Acid soluble	Native collagen	/	hMSCs	(29)
	Freeze-drying/EDC cross-linked	Scaffolds	/	Primary human and rat nasal septum chondrocytes and Rat septal cartilage defect model	(45)
	Freeze-drying/EDC cross-linked	Scaffolds	Alginate	hMSCs	(47)
	Enzymatical hydrolysis	Peptide	/	Human (clinic)	(109)
	/	Scaffolds	/	Rats	(45)
Dental tissue engineering	Enzymatical hydrolysis	Peptide	/	Rat odontoblast-like cells (MDPC-23)	(30)
	Enzymatical hydrolysis	Peptide	/	Primary human periodontal ligament cells	(31)
	Elecrospinning	Scaffolds	Bioactive glass/Chitosan	Primary human periodontal ligament cells and dog furcation defect model	(38)
Nerve regeneration	Enzymatical hydrolysis	Peptide solution	/	Rats	(53)
	Acid dissolution	tilapia collagen gel	/	hiPSCs	(37)
Skin tissue engineering	Enzymatical hydrolysis	Peptide solution	/	Rabbit	(14)
	Electrospinning	Nanofibers	/	Rats	(24)
	Hydrolyze	Peptide solution	/	/	(110)
	Solvent casting/Glutaraldehyde cross-linked	Scaffolds	/	L929 cells and Rat wound model	
	Freeze-drying/glutaraldehyde cross-linked	Scaffolds	/	Primary human fibroblasts and keratinocytes and Rat wound model	(78)
	Freeze-drying/Dehydrothermal treatment at 105°C	Scaffolds	Shrimp shell chitosan/glycerin	Primary human fibroblasts and keratinocytes	(35)
	EDC cross-linked	Scaffolds	/	NIH3T3 cells	(111)
	Freeze-drying/EDC cross-linked	Scaffolds	Alginate/Chitooligosaccharides	Primary human dermal cells	(79)
	Freeze-drying	Scaffolds	/	Hamster kidney fibroblasts (BHK21)	(112)
	Freeze-drying	Scaffolds	/	Rat wound model	(26)
	Freeze-drying/EDC cross-linked	Scaffolds	Chitosan	Mouse embryonic fibroblasts (MEF) and Rabbit wound model	(80)
	Enzymatical hydrolysis	Peptide	/	Human keratinocyte (HaCaT) and Rabbit scald wound model	(14)
	Enzymatical hydrolysis	Peptide	/	Rat wound model	(54)
	Electrospinning	Scaffolds	Bioactive glass	HaCaT cells, dermal fibroblasts and HUVECs	(36)
	Casting-solvent evaporation technique	Native collagen	/	Swelling behavior	
	Freeze-drying/ceftazidime cross-linked	Scaffolds	/	NIH3T3 cells	(113)
	Freeze-drying/Glutaraldehyde cross-linked	Scaffolds	/	NIH3T3 cells and HaCaT cells	(114)
	Electrospinning	Scaffolds	Chito oligosaccharides	Human skin fibroblasts	(115)
	Enzymatical hydrolysis	Peptide		Human (clinic)	(116)
	Enzymatical hydrolysis	Peptide	/	Human (clinic)	(117)
	Enzymatical hydrolysis	Peptide	/	L929 and HaCaT cells	(63)
Wound healing	/	Tilapia collagen extract	/	Rats	(24)
	/	Peptide solution	/	SD rats	(118)
	Enzymatical hydrolysis	Formulated into a cream	/	Male white rats (Rattus norvegicus)	(68)
	Electrospinning	Nanofibers	/	HaCaTs and SD rats	(39)
	/	Hydrogel	/	Albino rats	(119)
	Freeze-drying	Peptide solution	/	Mice	(44)
	Freeze-drying	Peptide solution	/	NIH3T3 cells	(72)
Corneal tissue engineering	Decellularization/Decalcification	Scaffolds	/	Rat ocular implantation model	(32)
	Drying at 25°C	Native collagen	/	Human limbal epithelial cells	(81)
Vascular tissue engineering	/	Peptide solution	/	CAVECs and Wistar rats	(120)
	Freeze-drying/Cold-pressing/1,4-butanediol diglycidyl ether cross-linked	Scaffolds	/	Mouse lymphatic endothelial cells	(67)
	Electrospinning	Scaffolds	PLGA	Primary rabbit aortic endothelial cells and smooth muscle cells	(40)
Oral mucosa regeneration	Freeze-drying/Dehydrothermal cross-linked	Scaffolds	Chitosan	Primary oral keratinocytes	(33)
Spinal cord injury repair	/	Double-layer collagen membrane	/	/	(121)

*hiPSCs, human induced pluripotent stem cells; hMSCs, human marrow stromal cells; BMSC, bone marrow stromal cells; HUVECs, human umbilical vein endothelial cells; CAVECs, carotid artery vascular endothelial cells*.

All of the fish collagen extracts were found to have high levels of imino acids (227-232/1000 residues). All collagen is soluble at acidic pH. In addition, the high collagen content, especially in the skin, and the good thermal properties [thermal transition temperature (31.6–33.7°C) and thermal denaturation temperature (31.1–32.2°C)] of the extracted collagen suggest that they have great potential as a collagen substitute in mammals (70). The low denaturation temperature of sponge collagen enables gelatin extraction at a lower temperature than that of mammalian gelatin. MDC is considered to be an equivalent biomaterial that is safer than the land-based biomaterials that currently dominate the market. The results showed that sponges A. cannabina and S. carnosus could be used as substitutes for collagen. If marine sponge is used as gelatin raw material in the food industry, it will bring high economic benefit (16). MDC also has promising applications *in vitro* 3D bioprinted models. But not the product of the modification of collagen and collagen denaturation gelatin easy rapid degradation. In order to solve this problem, in past research, scientists have developed a collagen and gelatin crosslinking of the chemical and physical methods, increasing the tunability of their mechanical properties. Marine collagen can be used as a biomaterial for tissue engineering and 3D bio-printing by controlling the content of methacrylate, as well as the intensity duration of ultraviolet light and the concentration of photoinitiator to control the required degree of cross-linking (122). The hydrogel with rheological characteristics was prepared by combining high-yield collagen with chondroitin sulfate. In addition, prionace glauca (PG) pepsin-soluble collagen (PSC) combined with shark-derived chondroitin sulfate produces a hydrogel with a cohesive polymer matrix that can be used for cartilage regeneration (91). The results showed that the best collagen yield was obtained when the papain concentration was 7,000 U/mg, and the pH value was 5.90, 22.79% collagen was hydrolyzed with alcalase and then separated by gel filtration chromatography. Compared with unhydrolyzed collagen, the four major components of the hydrolyzed product showed significant antioxidant and antiglycosylation activity (123). Based on previous studies, we summarized some characterization methods of MDC, aiming to understand the characteristics of the components of MDC (Table 1).

## MDC in Medical Tissue Engineering

**Figure 2:**
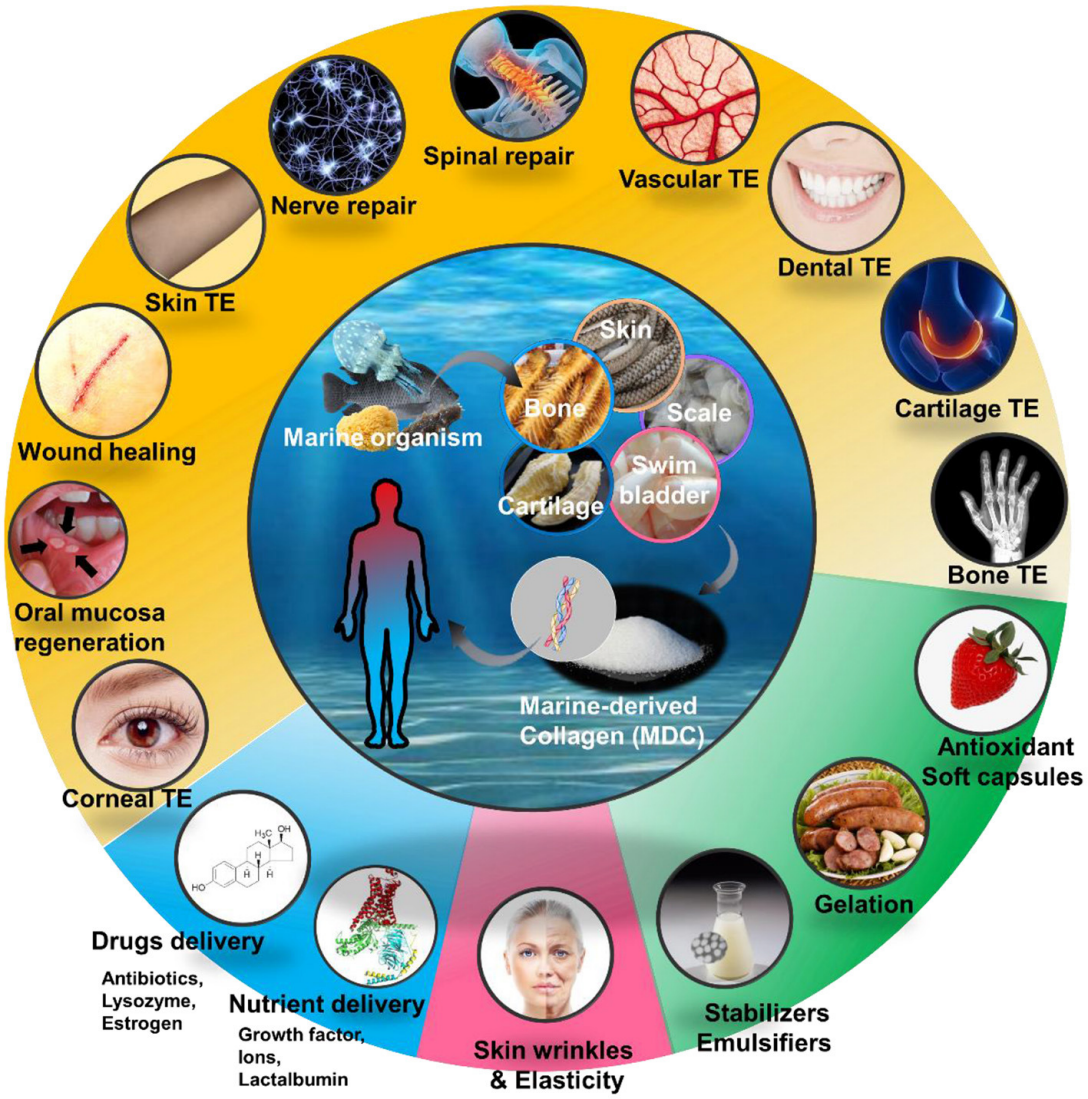
Applications of marine-derived collagen (MDC) for various area in human health, mainly including medical tissue engineering (TE), drug delivery system, cosmetics and Facials, and foods and health care products.

Tissue engineering and regenerative medicine is an emerging and rapidly growing life sciences domain. Using engineering and biological theory to create biomimetic tissues and organs on the basis of biological materials has become a common idea and hot topic among scientists in recent years. The excellent biocompatibility of MDC has stimulated its potential role in the design of biomaterial scaffolds in tissue engineering and regenerative medicine (Figure 2; Table 4).

### Bone Tissue Engineering

MDC with its high hydrophilicity and amino acids provides the optimal extracellular microenvironment and has many applications in bone tissue. It can promote the proliferation and differentiation of osteoblasts, and the bone marrow mesenchymal stem cells (BMSCs) that induce osteoblastic differentiation retain their immunomodulatory function. For example, tilapia collagen can promote the growth and differentiation of osteoblasts without the use of any additional induction reagents (28, 49), just as human bone marrow mesenchymal stem cells (hMSCs) readily adhere to tilapia squamous collagen during cell culture *in vitro*, thus significantly accelerating the early differentiation of hMSCs into osteoblasts (75). Biphase scaffolds of biomimetic mineralized salmon collagen and fibrotic jellyfish collagen were prepared by combining lyophilized and cross-linked methods, indicating that they can support chondroblast and osteogenic differentiation of hMSCs *in vitro* (41). Shark skin collagen also promotes the growth of osteoblasts and the synthesis of collagen in bone cells (49). When this collagen was further mixed with calcium phosphate from shark teeth to form a 3D composite scaffold, it could support the attachment and proliferation of osteoblast-like cells (48). Some researchers also found that the collagen peptide extracted from the scales of two kinds of fish, Sephareidae, can promote the proliferation of osteoblasts and inhibit the proliferation of mature osteoclasts, which can be used to prevent osteoporosis and help bone remodeling (50). In Codfish, low concentration of fish collagen peptide (FCP) may promote the proliferation of cells, and also promote the expression and differentiation of apoptotic osteoblasts (74). Collagen in salmon skin can also significantly up-regulate gene expression of various collagen-modifying enzymes in mouse pre-osteoblastic cells (MC3T3-E1) osteoblasts, which has a positive effect on osteoblasts (59).

MDC can also play a great role in bone development and bone injury repair. They used a sponge-collagen-based (SPG) scaffold and photobiodularization (PBM) to test a model of skull defect in Wistar rats. The results showed that SPG/PBM treated rats showed more connective tissue and newly formed bone tissue in the defect area (66). Mixing sponge collagen with hydroxyapatite (HA) to form scaffolds has the potential to improve graft performance for bone regeneration applications (62). MDC peptide (MCP) extracted from salmon skin was used to study the femur of growing rats. The results showed that MCP supplementation could increase the femur volume, bone density, dry weight and ash content of growing male rats. Therefore, MCP supplementation could promote the development of long bone in growing male rats (104): The effects of MDC oligopeptides and calcium aspartate on bone mineral density in ovariectomized Wistar rats were studied. It was found that the combination of MDC oligopeptides and calcium aspartate could significantly improve bone mineral density, which also indicated that MDC oligopeptides could promote the absorption of calcium aspartate (103). All these indicate that MDC has a good effect on bone growth and development.

In future studies, MDC may also provide new options for bone grafting and regeneration. Researchers have successfully developed a novel collagen fiber wikestone hydrogel based on the dual network (DN) concept using fish swim bladder collagen (SBC) extracted from sturgeon. The gel was implanted into the osteochondral defect of rabbit knee joint and showed good biomechanical properties *in vivo*. Mixed with hydroxyapatite wrapped DN gel combined with bone also is good. This kind of new collagen matrix composite DN gel has good biomechanical properties and combined with bone, is a kind of soft, elastic ceramic material, to design the next generation of orthopedic implants as artificial cartilage, the body weight bearing area of bone defect repair material provides a new choice (76). For bone regeneration, low immunogenicity fish collagen protein and bioactive nano-hydroxyapatite (N-HA)-reinforced polylactide glycogen (PLGA) nanofiber membranes were prepared for electrospinning guided bone regeneration (GBR). It was found that the membrane had good cytocompatibility with bone marrow mesenchymal stem cells (BMSCs) and human gingival fibroblasts (HGF). The experimental results showed that the composite fibrous membrane has great potential to guide bone or tissue regeneration (6).

### Cartilage Tissue Engineering

MDC plays a very important role in cartilage tissue and enhances chondroblast differentiation. For example, the researchers experimented with chondrogenic differentiation of human bone marrow mesenchymal stem cells (hMSCs) on the collagen fibers of tilapia scales and compared them with porcine collagen and uncoated culture dishes. The results showed that tilapia collagen fibrils in chondrogenic medium specifically enhanced chondrogenic differentiation of HMSCs. Therefore, collagen from tilapia scales can provide a suitable source of collagen for chondrogenic formation of hMSCs *in vitro* (29). Jellyfish collagen can also be used as a novel cartilage repair implant, using active growth factor nanoreservoir (TGF-β3), adult human mesenchymal stem cells derived from bone marrow. It was found that jellyfish type collagen implants led to chondrogenic differentiation of mesenchymal stem cells, and TGF-β3 as a nanoreservoir led to chondrogenic gene expression and chondrogenic differentiation (42). Using jellyfish collagen as a scaffold, for example, the researchers developed a three-dimensional porous scaffold with interconnected pores that can support and maintain chondrogenic differentiation of human mesenchymal stem cells (105). Porous scaffolds of jellyfish collagen fibers and sodium alginate hydrogels are also available (47). And it can stimulate the differentiation capacity of some other cells. For example, the researchers used the blue shark skin collagen with and without external stimuli induced human fat stem cells (hASC) their potential to differentiate into cartilage cells (71), and the sharks and rays of collagen combined shark chondroitin sulfate can be used to simulate human cartilage extracellular matrix. That suggests the MDC, a biomaterial, can be used as a template for cartilage regeneration (91). MDC stimulated the differentiation of chondroblasts and further promoted the formation of cartilage. The researchers prepared a three-dimensional porous fish collagen (FC) scaffold using MDC by freeze-drying technique. When rabbit auricle chondrocytes were implanted into porous fish collagen, it was found that it promoted the formation of chondrospecific extracellular matrix (ECM) *in vivo* and *in vitro*, and thus promoted the formation of cartilage under the rabbit skin (106, 107). TGF-β1 can induce chondrogenesis of adipocyte stromal cells (ADSCs) by adding fish collagen to TGF-β1, which can induce chondrogenesis effectively (108).

In cartilage tissue repair, the MDC matrix provides excellent performance for cartilage tissue engineering through the experiments of nasal cartilage repair with MDC *in situ* model of rats (45). It also has a protective effect on cartilage (77).

### Dental Tissue Engineering

In previous research, some researchers used type I collagen from tilapia scales in rat experiments to show that it has similar biocompatibility with pig skin collagen, which reminds us that tilapia scales collagen has the potential to replace mammalian type II collagen in oral and maxillofacial tissue regeneration. Soon after, the researchers carried out the periodontal membrane cell culture experiment of hydrolyzed tilapia collagen and proved that it had the function of periodontal tissue regeneration *in vitro*. The collagen of tilapia was extracted by electrospinning method, and the composite nanofiber membrane was prepared with bioactive glass and chitosan. The cell viability and osteogenic gene expression of human periodontal ligament cells (HPDLCs) were detected by the composite membrane in the canine class II bifection defect model experiment. It also promoted the expression of Runt-related transcription factor 2 (RUNX-2) and osteopontin (OPN) proteins (30, 31, 38). In conclusion, the application of MDC in teeth also has great potential.

### Vascular Tissue Engineering

MDC also has some applications in vascular tissue. For example, MDC can promote the growth of vascular endothelial cells. Experimental studies have investigated the protective effect of MDC peptides (MCPs) on carotid vascular endothelial cells (CAVECs) in type 2 diabetes mellitus (T2DM) and its mechanism. They injected Wistar rats with different concentrations of MCPs. *In vitro*, the vascular/endothelial construction of human umbilical vein endothelial cells (HUVECs) was cultured. Then, inflammatory exudation and related molecular markers of the vena cava endothelial cells were detected and analyzed. The results showed that MCP treatment for 4 weeks significantly reduced blood glucose, endothelial thinning. And inflammatory exudation of carotid vascular endothelial cells was reduced in rats. *In vitro*, high glucose intervention increased apoptosis in HUVECs significantly. Moderate and high doses of MCPs partially improved this high glucose mediated apoptosis and reduced the level of apoptotic biomarkers. Therefore, moderate dose of MCP inhibits apoptosis and reduces the expression of coupling factor 6 and microparticles, suggesting that we can use MCP to prevent early cardiovascular complications of T2DM (120). Some researchers also used freeze-drying and electrospinning to prepare MDC and PLGA fiber tubular scaffolds for vascular transplantation, and the electrospinning fiber PLGA layer on the surface of porous tubular collagen scaffolds in dry and wet states improved the mechanical strength of collagen scaffolds. The results showed that co-culture of smooth muscle cells (SMCs) and endothelial cells (ECs) using a collagen-PLGA scaffold under a pulsating perfusion system enhanced the development of vascular EC and preserved the differentiated cell phenotype (40).

Due to the good biocompatibility of fish collagen, the researchers use extra methylation modification and 1, 4-butanediol diglycidyl ether (BDE) crosslinking steps to improve the scales of the collagen derived from the physical and chemical properties. It was found that collagen integration plaques with the surrounding tissue was good. The infiltration of cells, blood vessels and lymphatic vessels was good. This study demonstrates the collagen derived from fish scales as a promising scaffold material in various biomedical applications (67).

### Spinal Cord Injury Repair and Nerve Regeneration

MDC has also been used in spinal cord regeneration. A new double-layer collagen membrane was designed and tested in a rat model of incomplete spinal cord injury. The previous research results showed that the transplantation of neural stem cells into a double-layer collagen membrane with different pore size promoted the differentiation of neural stem cells, alleviated the pathological injury, and improved the motor function of rats with incomplete spinal cord injury significantly (121).

Tilapia skin collagen was obtained by acid solution method and the stiffness of brain tissue was replicated for *in vitro* recombination experiments. By adding a cross-linker, a gel with a hardness similar to that of living brain tissue (150–1,500 Pa) was obtained, and the ability of the gel as a stem cell medium and the effect of hardness on neural lineage differentiation using human Induced pluripotent stem (iPS) cells were further investigated. It was found that exposure to a gel with a hardness of about 1,500 Pa promoted the production of neurons in the dorsal cortex during the early stages of neuroinduction (37).

To study the neuroprotective effects of MDC peptides (MCPs) isolated from salmon skin by enzymatic hydrolysis on perinatal asphyxia in male rats. Researchers found that MCPS promoted long-term learning and memory in perinatal asphyxia (PA) pups by decreasing oxidative damage and acetylcholinesterase (AChE) activity in the brain, and increasing the expression of p-CREB and brain-derived neurotrophic factor (BDNF) in the hippocampus (53).

### Skin Tissue Engineering and Wound Healing

MDC has significant biological activity and plays an important role in skin tissue. MDC can promote wound healing. For example, the study used the MDC peptide (MCP) in Nile tilapia skin to carry out the burn wound experiment in deep part thickness of rabbits and the scratch experiment *in vitro* of rats (14). At the same time, there is also a research team, for example, using porous collagen sponge to conduct experiments on burned wounds in rats (114), using jellyfish collagen polypeptide to conduct oral experiments in rats and salmon skin wounds in rats (54), using ethylene amine and fish scale collagen to conduct wound experiments in rats (113), all of which indicate that MCP can promote wound healing. Moreover, its suitability as a dermal substitute was found in the wound healing experiment of rat model (78). The researchers found that MDC could quickly and effectively promote the wound healing of rats (26). If it is made into scaffolds or nanofibers, it can also promote wound healing. The researchers prepared chitosan/sponge collagen/glycerin three-dimensional porous scaffolds and bionic electrospinning fish collagen/bioactive glass (COL/BG) nanofibers. The healing experiments on rat skin wounds also showed the ability of MDC to promote wound healing (35, 36). At the same time, MDC is also an excellent scaffold for skin tissue regeneration (79), and a potential wound dressing with antimicrobial properties (115).

*In vivo* experiments with MDC scaffolds from Cadfish and Weever showed that the scaffolds promoted the proliferation and migration of NIH/3T3 fibroblasts, and promoted tissue regeneration and healing (111, 124). Fibroblasts from small hamster kidney (BHK21) were inoculated on a three-dimensional collagen gel. The results showed that it could activate the proliferation of BHK-21 cells, so MDC could be used as a potential biomaterial extract for biomedical applications (112).

Hydrolyzed collagen is a kind of more and more popular health care products, its molecular weight is very low peptide, easy to be digested, absorbed and distributed by human body. Many clinical trials have been completed and current studies have shown the effects and benefits of collagen peptides on skin, such as hydration, elasticity and reduction of wrinkles. Therefore, hydrolyzed collagen can be considered an important weapon in the world every day in the fight against skin aging (116). Some researchers used hydrolyzed MDC to conduct experiments on the cheek skin of women aged 45–60 years old, and found that it could reduce skin wrinkles, enhance elasticity and tightness, improve gloss, and effectively improve the skin health (110). Orthosilicic acid, which hydrolyzes collagen and stabilizes it, which also has this effect (117).

MDC plays an important role in skin wound healing. Researchers used Nile tilapia skin collagen extract to promote skin wound healing in rats, and the experimental group showed obvious signs of skin healing. Moreover, the expression levels of vascular endothelial growth factor (VEGF) and transforming growth factor-β1 (TGF-β1) were significantly increased, and the gene expressions of VEGF, basic fibroblast growth factor (bFGF) and Alpha-smooth muscle actin (α-SMA) were significantly up-regulated. These results indicate that local application of Nile tilapia collagen extract can promote skin wound healing in rats, which may be due to its stimulating effect on the recruitment and activation of macrophages to produce chemotactic growth factors, fibroblast proliferation and angiogenesis (24). The researchers also used Snakehead fish collagen, Queen Fish skin collagen, Rhizostoma pulmo jellyfish collagen, and Giant Croaker (Nibea japonica) swim Bladders Collagen Japan swim bladder was used for wound healing experiment. Results show that the snakehead ossein paste made of white male rats sewer rat wound healing the best dose of 3% concentration (68), preparation of fish skin collagen hydrogel promote epithelial regeneration, and no water gel processing rat inflammatory cells angiogenesis, collagen deposition and hexose amine content, epithelium and wound contraction increased significantly (119). At the same time, Jellyfish collagen promotes artificial wound formation on the monolayer of human umbilical vein endothelial cells (HUVECs) (44). Japanese loach swim bladder Acid-soluble collagen (ASC) and pepsin soluble collagen (PSC) have good application in wound healing of mouse *in vitro* scratches (72). The researchers also found that oligopeptide compounds derived from marine fish peptides (MFPs) have the potential to significantly increase uterine scar tension, reduce the risk of uterine rupture, and promote uterine wound healing in rats following cesarean section (CS). It is speculated that its promoting effect may be related to the formation of new capillaries in scar tissue, the growth and repair of collagen fiber and smooth muscle tissue (118).

### Oral Mucosa Regeneration

MDC also plays a role in the repair of oral mucosa. Researchers prepared chitosan-collagen composite scaffolds (C3) to construct oral mucosal equivalents (EVPOME-C) *in vitro*, and compared EVPOME-C with oral mucosal equivalents (EVPOME-B) and natural oral mucosa constructed with Alloderm® (EVPOME-A) and Biomend®. The results showed that the C3 scaffold has a well-developed fiber network and a small enough porosity to prevent keratinocytes from growing in the scaffold after cell inoculation. The C3 scaffold has potential application value in epithelial tissue engineering, and provides a new treatment method for oral mucosal regeneration medicine (33).

### Corneal Tissue Engineering

MDC has also been used in corneal tissue, in which fish scale-derived collagen matrix (FSCM) has been proposed as a substitute for human donor corneal tissue. To assess its biocompatibility, the FSCM was implanted as an anterior lamellar keratoplasty (ALK), placed in the interlamellar pouch (IL) and placed in the subconjunctiva (SC). The light transmittance was found to be similar to that through the human cornea. Implanting FSCM as an ALK resulted in only mild blurring, not pupil blurring, despite the presence of new blood vessels around the sutures; Interleukin placement causes moderate haze, partial occlusion of the pupil, and (partial) anterior lamella melting. The SC group showed local swelling and sclerosis, which decreased over time. Histology showed mild to moderate chronic inflammation in the ALK and IL groups, while severe inflammation was found in the SC group. Despite the technical difficulties, treatment of ALK with FSCM is feasible, while IL placement can cause anterior lamina melt. Further studies are needed to better understand its immunogenicity. The light scattering and transmission data suggest that the first version of the FSCM is comparable to human corneal tissue in this respect (32).

## MDC in Drug Delivery System

MDC plays an important role in the drug delivery system, as shown in Table 5. For example, the researchers report a simple method of preparing collagenous peptide-chelated calcium (CPCC) from marine fish scales and a novel CPCC-loaded nanoparticle to supplement calcium. Their experiments showed that core-shell CPCC significantly increased bone mineral density and calcium content in the femur of rats, so the CPCC and core-shell CPCC nanoparticles are ideal choices for calcium supplementation (46). Acid-soluble collagen (ASC) and pepsin soluble collagen (PSC) isolated and identified from the waste skin of sea eel (Evenchely smacrura) can also be used for *in vitro* drug release experiments (83). The naturally keratinized sponges (Porous fungi, Dictyoceratida) are high in glycosaminoglycan content. It can be administered topically as a bio-based dressing and a biological active bionic carrier to regulate the process of wound healing (84). There are also spongy renal cartilages. A water-based gastric acid resistant coating dispersion was developed using renal sponge collagen 15% (W/W) as film forming agent. The results showed that the sponge collagen was resistant to drug for more than 2 h under the action of 0.1 M hydrochloric acid and disintegrated within 10 min in the phosphate buffer solution of pH 6.8. The coated tablets had good mechanical properties and could be stored for more than 6 months without loss of intestinal solubility (60). In hormone replacement therapy, transdermal administration of estradiol bypasses the liver system before metabolism, and therefore has better side effects than oral estrogen. Renal cartilage sponge collagen nanoparticles were used as an osmotic accelerator for transdermal delivery of 17β-estradiol-hemihydrate for hormone replacement therapy. The results showed that the hydrogels containing estradiol collagen nanoparticles could prolong the release time of estradiol and significantly improve the absorption of estradiol. Therefore, sponge collagen nanoparticles are a promising carrier for transdermal drug delivery (64).

**Table 5:** Applications of MDC in drug delivery system.

**Loaded drugs**	**Forms**	**Additive materials**	**Biological evaluation model**	**References**
Antibiotic (ampicillin and tetracycline)	Powder and film	/	/	(64)
Lysozyme	Microparticles	/	/	(46)
Growth factor (bFGF)	Scaffolds	Chitosan/chondroitin sulfate/PLGA	Rat full-thickness skin wound model	(82)
Ion (Calcium)	Nanoparticle	Calcium alginate	Rats' femur	(83)
Ion (Calcium)	Injectable gel	Chitosan	Rats	(60)
α-lactalbumin	Microparticles	/	/	(46)
Estrogen (17-beta-estradiol-hemihydrate)	Nanoparticle	/	Postmenopausal women	(27)
Gastroresistant tablets	Enteric coating	/		(65)

The researchers mixed MDC with other biomaterials. The chitosan and chum salmon skin MDC composite gel materials. The compound gel was injected subcutaneously into the back of rats. The specimens were collected for histological examination and ELISA to detect tumor necrosis factor α (TNF-α). It was found that the composite gel could be used as a carrier of tissue filler and drug delivery system (65).

MDC also has potential as a microprotein delivery system. The microgranular protein delivery system was developed using collagen extracted from the jellyfish Catostylus tagi as a polymer matrix. The researchers extracted collagen microparticles by emulsification-gel-solvent, and the CMPs collagen microparticles was cross-linked with 1-ethyl-3-(3-dimethylaminopropyl) carbon diimine (EDC). *In vitro* experiments showed that cross-linking also resulted in greater stability of CMP in water, allowing for slow release of microgranular proteins. These show the potential use of MDC in the production of microparticles for the controlled release of therapeutic proteins (27).

## MDC in Cosmetics and Skincare

MDC is a good moisturizer candidate, which has a wide range of functions in cosmetics. The researchers used MDC from the skin of salmon and cod as an ingredient in cosmetic formulas. Then the experimental results showed that collagen exhibited good water retention ability. Therefore, it is suitable as a moisturizer for skin application. Molecular markers of irritation and inflammation were analyzed that local exposure to collagen in the reconstructed human dermis was found to have no stimulating potential (85). The researchers also isolated collagen from grouper swim bladders and turned it into nanoscale collagen. To determine whether the chemical composition of collagen meets the quality standards of cosmetic raw materials, they did a lot of experiments. Finally, they found it have met the quality requirements of collagen standards as a cosmetic material based on Standar Nasional Indonesia (SNI) (87).

## MDC in Foods and Health Products

MDC is also widely used in the field of food science and health products (Table 6). Currently, MDC or other-sourced collagen can be used as an emulsion to modify food, such as fish oil. Fish oil is rich in omega-3 unsaturated fatty acids and has many important physiological functions and potential for disease prevention. However, there are many disadvantages about it, such as its double bonds are too unstable to rupture, its fishy taste, and poor water solubility. These limit the application. There is a need to develop new formulations, food-emulsions are a practical method, to encapsulate fish oils for protection, increase water solubility and isolate the fishy smell.

**Table 6:** Applications of MDC in foods and health care products.

**Applications**	**Forms**	**Functions**	**References**
Emulsion	Gelatin	Emulsion in food industry.	(125)
	Gelatin	Fish oil-loaded gelatin-stabilized emulsions in food.	(126)
	Gelatin	Optimal emulsion storage and transportation conditions in food.	(127)
	Gelatin and peptides	Decrease the creaming stability	(128)
Gelation	Peptides	Gelatins can increase the droplet stability and effect on the phase transition.	(129)
Antioxidant	Peptides	GPEGPMGLE, EGPFGPEG, and GFIGPTE, might serve as potential antioxidants applied in nutraceutical and pharmaceutical products.	(130)
	Gelatin	Antioxidative MCPs may increase life span and protection against tumor development.	(131)
	Peptides	Peptides serve as natural antioxidants in food and cosmetics.	(132)
Soft capsules	Gelatin	Electrospun nanofibers of MDC transport fish oi or nutrients to the stomach and intestines.	(133)

There are many influence factors in the use of food-emulsions. To improve the emulsion's stability, researchers need to keep our eyes on the temperature, pH, surface modification, storage time and so on. Emulsion stability mainly depends on droplet size and shell thickness (134). Higher storage temperatures (4–37°C) cause the fish oil emulsion to change from a liquid form to a redispersible gel form. It shows that increased temperatures decreased the creaming stability differences (128). The pH of gelatin solution, the speed of homogenizer and the homogenizing time also have important effects on the stability of the emulsion. The gelatin solution pH, speed of homogenizer and the homogenizing time also have important effects on the stability of droplet sizes linearly decreased with increased of solution pH and homogenizing times. Droplet sizes exponentially decreased with increased of homogenizing speeds (135). There are results demonstrating that Cooperative adsorption has better emulsion stability than competitive adsorption. In the work, they mainly explored the gelatin is combined with four surfactants [soybean lecithin (SL), sodium dodecyl sulfate (SDS), Span 80 and Tween 80], which adsorb each other at the oil-water interface, which can improve or decrease the stability of the emulsion (136). These results are connected with the changes of pH, too. The stability results of gelatin/surfactant co-stabilized (Span 80 and SL) or competitive stabilized (Tween 80 and SDS) were studied under different pH backgrounds (137).

Gelations can be modified by different surface modifications. Bovine and fish gelatins were modified by octenyl succinic anhydride (OSA) (125). The DS increase of OSA-modified bovine bone gelatins increases the droplet stability, but the DS increase of OSA-modified fish skin gelatins can only increases of the droplet stability and effects on the phase transition and creaming index of fish oil-loaded emulsions is very weak. The new formulation of oat β-glucan (OG)-MDC peptide mixed gels was researched. It has guiding significance for the formulation of low-fat meat products and is beneficial to improve food safety and nutritional value (129).

Collagen peptides may be used as a potential antioxidant in nutritional and pharmaceutical products. Prious research has shown that collagen peptides can serve as natural antioxidants in a variety of applications, such as food and cosmetics (138). Antioxidant Peptides from Gelatin Hydrolysate of Skipjack Tuna (Katsuwonus pelamis) might serve as potential antioxidants applied in health food industries (130). Antioxidant peptides from collagen hydrolysate showed that collagen peptides might serve as potential antioxidants applied in nutraceutical and pharmaceutical products (131). Previous studies have shown that MDC-prepared skin has two effects, namely extending the life span of rats and inhibiting the spontaneous occurrence of tumors. This result indirectly proves that the antioxidant properties of MCPs may be the cause, regarding the extension of life and protection of tumor development (132).

## Conclusion and Outlook

Marine-derived collagen (MDC) has good biocompatibility and biodegradability. In recent years, scientists have made extensive exploration in food emulsions and biomedical applications. MDC can be extracted from fish waste products, which is an economical and sustainable source of collagen and can be used as an alternative to land-based collagen. Land-based collagen carries the risk of transmission of zoonotic diseases such as bovine sponge encephalopathy and hand, foot and mouth disease. For religious reasons, pig-derived collagen cannot be used in some foods. MDC protein has a very important application in food. MDC can be used as a food emulsion to encapsulate fish oil for protection. It has guiding significance for the formulation of low-fat meat products and is beneficial to improve food safety and nutritional value. In nutraceutical and pharmaceutical products, MDC might serve as potential antioxidants, even can inhibit the development of tumors.

Similar to materials such as polyhydroxyalkanoate (PHA) (139), PLGA (140), MDC is widely used in medical tissue, especially in bone tissue engineering, cartilage tissue engineering and functional repair of skin tissue. Good biocompatibility makes it the best template for cell growth. At the same time, scaffolds made of MDC can enable cells to live in 3D space (141, 142), thus improving the efficiency of culture, and collagen can induce cell differentiation in some specific environments, so as to produce specific functions. In addition, due to its biodegradability, MDC can be a good drug encapsulation and sustained-release system (141, 143) to improve the effectiveness of drug delivery. Of course, MDC also has some drawbacks. MDC is not strong enough, which makes its scaffold mechanical properties inadequate. In 2015, tilapia was proved to have good biocompatibility and can effectively induce skin regeneration (25). In order to explore the potential clinical application value of gill dolphin collagen materials, gill dolphin collagen extracted from gill dolphin skin was compared with tilapia collagen (144). The gill dolphin collagen and tilapia collagen were dissolved in 0.5 mol/L acetic acid to form the membrane by casting method. The morphological structure, aqueous solubility and mechanical properties of gill dolphin and tilapia collagen membranes were characterized. The degradability and biocompatibility of the two materials were tested by subcutaneous implantation and cell culture (145, 146). The samples were detected at the experimentally specified time, and the application potential of the gill dolphin collagen membrane was evaluated by contrast with the tilapia collagen membrane. However, MDC also has the characteristics of low mechanical strength and rapid degradation *in vivo*, which can be solved by crosslinking with other natural or synthetic polymers. Therefore, 25% glutaraldehyde cross-linking can improve the mechanical strength and degradation characteristics of collagen membrane (147, 148). The residual glutaraldehyde after crosslinking was treated with glycine (149).

Based on this review, there are not many kinds of MDC available in the market at present, but there are abundant kinds of marine organisms with excellent physical and chemical properties. Therefore, the application prospect of all kinds of MDC is broad. As a new type of biomaterial, MDC egg has been widely recognized and attracted more and more attention from researchers in clinical, medicine, food and other fields.

## Author Contributions

NX, X-LP, and H-RL reviewed the literature and wrote this manuscript. J-XL and J-S-YC collected the data and critically reviewed this manuscript. X-YQ and S-JY reviewed the literature and wrote this manuscript. H-LG and X-HZ read and approved the final manuscript. JY and GX designed this manuscript. D-XW designed, reviewed the literature, and wrote this manuscript. All authors read and approved the final manuscript.

## Conflict of Interest

The authors declare that the research was conducted in the absence of any commercial or financial relationships that could be construed as a potential conflict of interest.

## Publisher's Note

All claims expressed in this article are solely those of the authors and do not necessarily represent those of their affiliated organizations, or those of the publisher, the editors and the reviewers. Any product that may be evaluated in this article, or claim that may be made by its manufacturer, is not guaranteed or endorsed by the publisher.
